# A national survey of current discharge planning and aftercare arrangement practices for those returned to prison from secure psychiatric services in England and Wales

**DOI:** 10.1192/bjo.2025.26

**Published:** 2025-05-13

**Authors:** Sarah Leonard, Jana Bowden, Matilda Minchin, Ruth McDonald, Neil Allen, Jane Senior, Jennifer Shaw

**Affiliations:** Centre for Mental Health and Safety, University of Manchester, Manchester, UK; NIHR ARC NENC Multimorbidity, Ageing and Frailty Theme, Faculty of Medical Sciences, Newcastle University, Newcastle upon Tyne, UK

**Keywords:** Mental Health Act 1983, secure services, prison mental health services

## Abstract

**Background:**

Little is known about the transition process for those returned to prison following treatment in secure psychiatric services. This study is the first internationally to explore the process of discharge/aftercare planning for this population.

**Aim:**

To identify the current national discharge and aftercare planning procedures for people returned to prison in England and Wales.

**Methods:**

A national survey of current service discharge planning and aftercare arrangement practices in low- and medium-secure psychiatric services and prison mental health teams in England and Wales.

**Results:**

We had a 72% response rate across prison-based and secure mental health services. A summative contents analysis highlighted that outstanding priority areas for improvement, include (a) relationship building to improve communication and understanding between secure psychiatric services, prison mental health services, and the prison estate and (b) significant reform and additional resources to achieve the expected standard of care and to provide people returned to prison with a care package tailored to meet their specific needs.

**Conclusion:**

Effective care planning and management of return to prison from secure psychiatric services has the potential to improve patient health and well-being in prison and up to and beyond their subsequent prison release, with far-reaching effects on prevention of relapse, hospital readmission, reoffending and other adverse events.

In England and Wales, prisoners who require in-patient mental health assessment/treatment, and who meet the criteria for treatment in hospital under the Mental Health Act 1983 (MHA), are referred for access assessment for admission to secure psychiatric services (SPS). These referrals can be directed by either clinicians within the prison mental health team (PMHT) or the criminal courts for assessment to inform sentencing decisions. SPS operate at three levels: low, medium, and high, with a patient’s risk determining the degree of security they will receive. Upon admission, while individual treatment plans will vary, the remit of SPS is assessment and/or treatment of mental disorder, management of the risks posed by a patient to others and prevention of subsequent reoffending.^1^ Continued liaison between the PMHT at the transferring prison and the SPS should be maintained from admission to ensure that progress, treatment and any discharge plans are managed, and all parties informed.^2^ Suitability for discharge from SPS is assessed by the patient’s responsible clinician and should be dependent on reduced need for treatment and supervision, and evidence of a reduction of risk.^3–4^

## Discharge from secure psychiatric services

Discharge destination options available to responsible clinicians are restricted by a patient’s legal and MHA status. For example, patients on remand may be sentenced during their admission by criminal courts to a hospital treatment order in lieu of a custodial sentence. Patients admitted to SPS during a custodial sentence may also have their sentence lapse during admission, resulting in their MHA status changing to a ‘notional hospital order.’ In both instances, discharge options are via a community care pathway only. Patients suitable for discharge from SPS but who have a remaining sentence tariff or are still awaiting trial/sentencing are returned (remitted) to prison. Return to prison may be requested under sections 50, 51 or 53 of the MHA if the responsible clinician advises the Secretary of State for Justice (via the Mental Health Casework Section of the Ministry of Justice) that the patient no longer requires treatment for their mental health needs, or that no effective treatment is available in the hospital where the patient is detained.^2^ Alternatively, if the first tier tribunal concludes that, under s.47 (transfer to hospital of sentenced prisoners) of the MHA a transferred patient would be entitled to a discharge if they were a restricted-hospital order patient, then the hospital managers may return them to prison, subject to any comments made by the first tier tribunal and the decision of the Secretary of State for Justice.

Planning and delivery of treatment in SPS is guided by the care programme approach (CPA), a framework used to assess the needs of each patient and ensure that appropriate support is in place to meet their needs.^5–6^ Planning for discharge is a priority within this framework, with particular emphasis on arrangements for aftercare services, designed to reduce the likelihood of deterioration and readmission. Those subject to Sections 45A (hospital and limitation direction), 47 (transfer of sentenced prisoners) and 48 of the MHA (transfer of prisoners on remand) have the right to ongoing aftercare following remittal to prison (s.117 of MHA). National Health Service (NHS) England and the local authority are jointly responsible for s.117 aftercare while the person is in prison. Entitlement to s.117 aftercare should be attended to in the same way that it would be in the community, apart from any provisions that are disapplied in a custodial setting (Care Act, para. 17.6-7). It is the responsibility of the SPS and representatives from the local authority to hold a s.117 discharge planning meeting to develop a CPA care plan. This should be held prior to return to prison (unless there are exceptional circumstances) and should be attended by the receiving prison PMHT and prison/operational staff.^2^ Detailed information on management on risk and treatment while the patient has been in hospital must be provided at this meeting, and any information relevant to care provision once returned to prison, ensuring that all treatment plans, needs and requirements, along with risks, are shared and agreed. Aftercare should last as long as there is a need to be met and must remain in place until such time that both the PMHT (or Independent Commission Board (ICB) if the person has been released into the community) and local authority are satisfied that the patient no longer has needs that require aftercare services. Individuals being released from prison and who have a s.117 aftercare entitlement should be referred by the PMHT to the relevant ICB and local authority as soon as practicable, in order to facilitate maximum opportunity for their s.117 aftercare plan to be updated prior to release.^7^

**Fig. 1:**
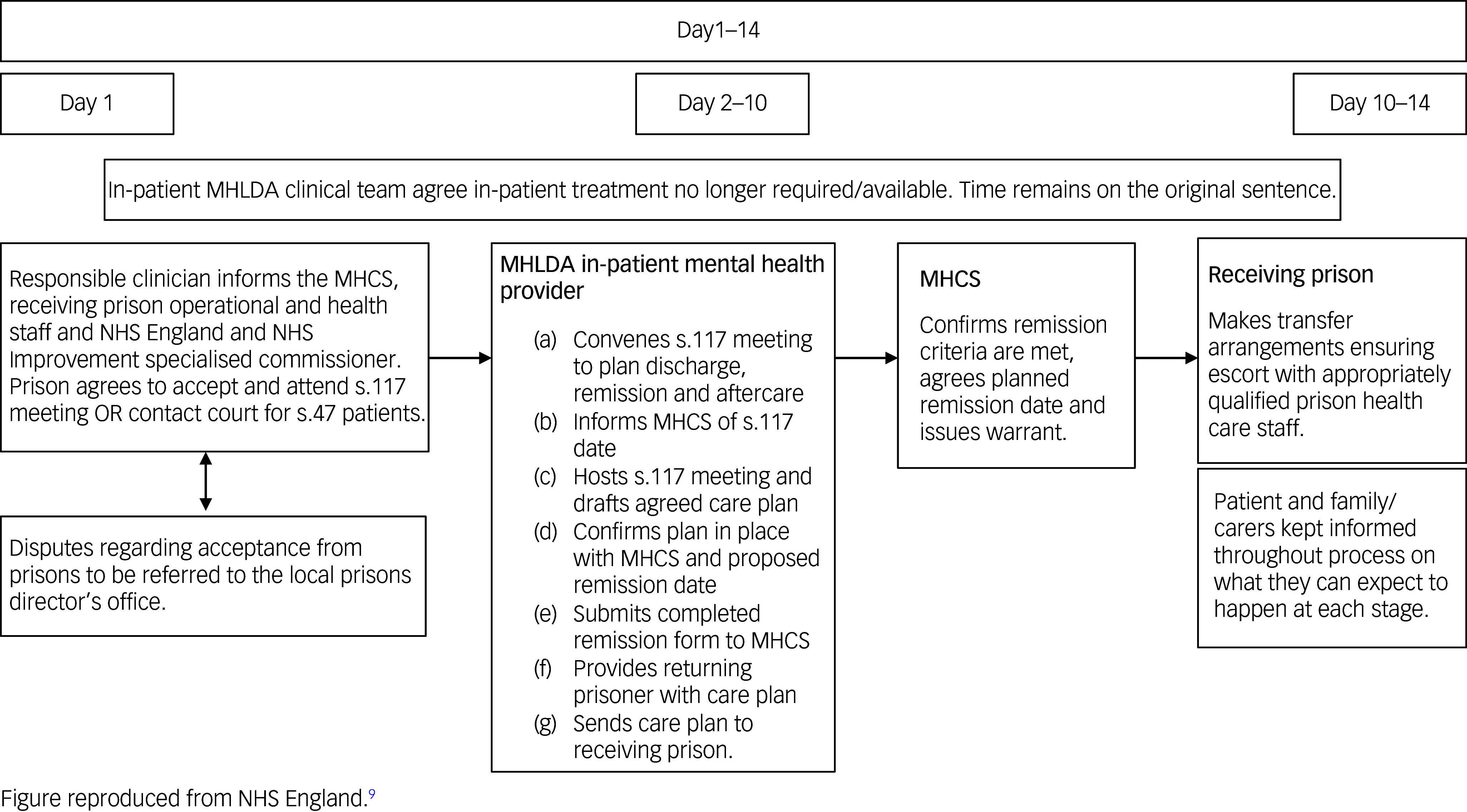
Transfer and remission of adult prisoners under the Mental Health Act 1983. Good practice guidance 2021: prison remissions and time limits. MHLDA, Mental Health, Learning Disability and Autism; MHSC, Mental Health Casework Section.

Guidance for remittal was first outlined in 2005 within the Offender Mental Health Care Pathway. Since then, there has been no formal evaluation of remittal guidance.^8^ In 2021, NHS England published updated good practice guidance which set out the process for transferring and remitting patients in England to and from prisons.^2^ This guidance introduced a remittal pathway time scale of a maximum of 14 days between a responsible clinicians decision to remit and transfer to the local prison establishment (or long-term and high-security estate for category A prisoner) and outlined the responsibilities assigned to the discharging SPS and receiving PMHT (see Fig. 1). This update does not, however, include guidance on effective planning for, and delivery of, aftercare following return to prison.^9^

## The current context

Returns to prison now constitute one in five of all discharges from SPS in England and Wales.^10^ Despite this, there is little research on the process of discharge planning, provision of aftercare and outcomes following return to prison. This group represents a complex subset of SPS patients who are particularly vulnerable at the time of discharge in comparison with those discharged via a community care pathway.^10–12^ To date, the only investigation to have examined the remittal care pathway consists of a national cohort study of a population of patients discharged from all 33 NHSmedium SPS.^11,12^ This study observed that those who had returned to prison experienced a significantly shorter length of stay in medium SPS as compared with those discharged into the community, with over half having a stay of 6 months or less. Investigation into clinical presentation prior to discharge observed that those who had returned to prison displayed significantly more issues with psychological adjustment at the time of discharge. Prison returners also presented as having a higher risk of future violence and a lower prevalence of protective factors that mitigate subsequent risks of relapse and reoffending.^12^ These findings suggest that the clinical threshold for appropriate discharge to prison is lower than that applied to those discharged into the community. This investigation also documented discharge circumstances that would not be acceptable for those discharged into the community, where over a quarter of patients were returned to prison owing to not engaging with treatment or being too ‘high risk’ to remain detained within the service.^11^

Follow-up of this cohort was conducted across 63 prisons, where it was observed that less than 20% of patients with legal entitlement to s.117 aftercare had their care guided by the CPA approach at any point during the 1 year follow-up period.^12^ While the majority of those remitted were referred to the PMHT (98%), only 75% were accepted on to the PMHT caseload. Non-acceptance was due to not meeting the PMHT criteria, as determined by the assessor/team. Of those accepted, 10% were discharged from the service during follow-up owing to either non-engagement or not meeting the PMHT criteria. In no instance of loss of aftercare was there documentation of formal discontinuation as outlined under s.117 of MHA. In the absence of robust aftercare, the study observed discontinuation of psychiatric medication against clinical advice (47%), use of the Assessment, Care in Custody and Teamwork care plan process (a care-planning process in custodial settings for those identified as being at risk of self-harm and/or suicide, 49%), use of segregation for behaviour management (21%), admission to healthcare wing for psychiatric care (37%), and referral (21%) and readmission (11%) to SPS, within the 1 year follow-up period. For those released into the community during the follow-up period, less than a half were referred to community mental health services (CMHS) prior to release. Preparing for release from custody poses a challenge for PMHTs, and it is well known that not connecting with an appropriate CMHS following release is associated with a higher risk of mortality, relapse, violence, reoffending, suicide and other causes of death.^13–16^

Collectively, these findings suggest that there is a current practice of premature remittal from SPS and/or lack of coordination with PMHTs and local authorities in ensuring receipt of adequate aftercare. Prisoners are entitled to a level of healthcare equivalent to that provided to the general population, and should be treated by services that are of the equivalent standard.^17^ We assert that this should extend to equivalent quality of discharge/aftercare planning for those returned to prison. Transition from SPS is a time of elevated risk, and it is therefore essential that these processes are as diligent as for those who are discharged into the community.

To understand how to improve the remittal care pathway, further evidence is needed regarding barriers and facilitators to safe and effective return to prison and aftercare provision. The present study aimed to identify the current national discharge and aftercare planning procedures for people returned to prison in England and Wales, via a national survey of SPS and PMHTs.

## Method

### Design

A national survey of current service discharge planning and aftercare arrangement practices in SPS and PMHTs in England and Wales. This design was selected because there is wide variation in resources available to manage SPS/PMHTs, and many different styles of service delivery exist. To gain an overview of the current processes and procedures that guide remittal to prison from SPS, a national survey was considered most appropriate.

### Ethics

The authors assert that all procedures contributing to this work comply with the ethical standards of the relevant national and institutional committees on human experimentation and with the Helsinki Declaration of 1975 as revised in 2013. All procedures involving human subjects/patients were approved by the Health Research Authority (REC reference: 20/HRA/5495).

### Sample and recruitment

All eligible SPS and PMHTs were contacted to take part in the surveys. Eligible SPS were high, medium and mixed (low and medium) SPS (*n* = 52). Eligible prisons were those identified as routinely receiving remitted patients from SPS (*n* = 60). Surveys were completed by clinicians who oversee the prison remittal pathway in each service.

### Materials

An inventory of procedures and practices was developed through the extraction of recommendations from: the Mental Health Act 1983 and Code of Practice 20151,^18^ ‘*The Good Practice Guide. The Transfer and Remission of Adult Prisoners under s47 and s48 of the MHA*’^2^ and the ‘*Royal College of Psychiatrists Quality Network Standards for Forensic Care and Prison MH Services CCQI*’.^19^ This inventory was used to develop the survey questions, under the guidance of two clinical members of the team who have practised in both PMHTs and SPS (J. Shaw and J. Senior). This included a breadth of closed questions that addressed the day-to-day processes involved in discharge/aftercare planning for prison remittals, alongside the opportunity to provide free text responses. The questionnaire planned to ascertain current discharge planning procedures for prison remittals and was designed to identify how well multi-agency services are integrated. As such, respondents were also invited to share insight into discharge planning and the aftercare process, including ‘what currently works well, and how the process could be improved’, in the form of free text responses. Space was provided for insights into the topics listed in Table 1.

**Table 1:** Topics proposed for free text insights

Topic	Prompt
(a) s.117 meetings	Invitation of prison mental health team (PMHT) by secure psychiatric services (SPS) and PMHT attendance
(b) Information handover	The nature and breadth of information handed over
(c) Aftercare planning	Joint planning between SPS and PMHT, resources available
(d) Care programme approach (CPA)	Completion of CPA by SPS, involvement of PMHT
(e) Discontinuation of aftercare	The formal process of discontinuing aftercare in prison

### Procedure

The questionnaire was distributed electronically via email to lead clinicians at each site between March 2021 and June 2022. In PMHTs, the team manager was invited to complete the survey; at SPS the clinical director was asked to complete the survey or delegate to the most appropriate member of their team. Leads at both site types were identified via a liaison exercise at each site to ensure that the request was made to a professional who has insight and operational understanding of local remittal care pathway procedures. The landing page of the electronic questionnaires contained the participant information sheet followed by a series of consent statements that clinicians were required to complete. Clinicians were asked to return the completed questionnaire to the research team via email. Following initial circulation, sites were recontacted three times via email with reminders of invitation and a telephone call offered should the site require further information. Where questions were unanswered or answers were ambiguous, sites were recontacted for clarification. Once returned, responses were anonymised prior to data inputting.

### Analysis

Numerical data were analysed using Statistical Package for the Social Sciences (SPSS) for Windows version 24 and descriptive statistics were generated. Free text responses were entered into NVivo analytical software to facilitate coding. The focus of the free text response analysis was not to develop emergent themes, but to identify key barriers and facilitators at each stage of the discharge planning and aftercare process. As such, the data for each free text response were analysed separately following a summative approach to content analysis.^20^ This allowed for identification of shared experiences via manifest content analysis,^21^ and, where explicit, surface-level content in the data was identified and labelled. This method focuses on tangible and directly observable content rather than underlying meanings or interpretations. Latent content analysis^22^ was utilised to interpret the data, this approach being more subjective than manifest coding, focusing on what is implied rather than explicitly stated. Manifest coding was derived by the content of the responses, where data were read for recurring key terms or content and then structured by ‘positive (facilitators)’, ‘negative (barriers)’, ‘neutral’ or ‘unrelated’. ‘Unrelated’ data were discarded unless the response was misplaced and belonged in a difference response section. Manifest coding was conducted by simultaneously two of the authors (J.B. and M.M.), with 50% cross-over to monitor consistency. Consistency and cases of ambiguity were monitored by S.L. Data were then latently coded to capture the inferred ideas, or meanings, rather than just the explicit content, followed by interpretation of the underlying context, where data were summarised narratively by S.L. Narratives were then circulated to the wider team for consideration. Verbatim quotes are used to illustrate key identified barriers and facilitators in Tables 4–7.

## Results

Survey responses were returned by 65% of SPS contacted for participation; two high SPS (9%), eight medium SPS which (23%) and 24 SPS which providing both medium- and low-secure care (70%). The majority of respondents indicated that their SPS has a defined local care pathway for those remitted to prison (n = 22, 65%); for 29% there was a local policy in place to support this, whereas for 35% this was an informal care pathway. Eleven (32%) services had no remittal pathway and were not in the process of developing one. A dedicated health and justice team/lead was reported as present at eight SPS (24%). Respondents were asked to indicate the importance of follow-up by SPS for post remittal: 79% indicated that this is either ‘very important’ (*n* = 15) or ‘important’ (*n* = 13), while 11% indicated that this is of ‘low importance’ (*n* = 4). Respondents were asked to indicate how well their local PMHT perform in their navigator function to coordinate patient care and ensure smooth pathways into and out of prison: 44% indicated that their PMHT performs either ‘not very well’ (*n* = 12) or ‘not well at all’ (*n* = 3), whille 35% indicated that it performs ‘fairly well’ (*n* = 10) or ‘very well’ (*n* = 2). See Table 2 for full SPS responses.

**Table 2:** Characteristics of secure services

Topic	*n* (%)
Security	
Medium-secure services	7 (20.6)
Combine medium- and low-secure services	24 (70.6)
High-secure services	3 (8.8)
Define remittal care pathway	
Care pathway present and there is a policy in place to support this	11 (32.4)
Care pathway present but this is informal	11 (32.4)
No defined care pathway	11 (32.4)
Don’t know	1 (3.0)
Dedicated health and justice team	9 (26.5)
Transfer coordinator	17 (50.0%)
How well are prison mental health services meeting this responsibility?	
Very well	2 (5.9%)
Fairly well	10 (29.4%)
Moderately well	6 (17.6%)
Not very well	13 (38.2%)
Not well at all	3 (8.8%)
How important do you think follow-up is from secure services?	
Very important	14 (41.2%)
Important	13 (38.2%)
Neutral	3 (8.8%)
Low importance	4 (11.8%)
Not at all important	0 (0.0%)

Survey responses were returned by 78% of contacted PMHT sites. Twenty-three PMHTs (49%) reported that the PMHT was the same provider as their local SPS. All PMHTs reported routinely asking those referred to their service about any previous psychiatric detention and routinely investigating whether a remitted person is subject to the care programme approach CPA. Seven PMHTs (13%) also reported contacting the patient’s community mental health team to discuss their CPA. Eleven PMHTs (23%) reported having a dedicated aftercare lead for those returned to prison from SPS; in all services this role was conducted by a nurse. Thirty-two PMHTs (68%) reported having no aftercare lead. Respondents were asked to indicate how well their PMHTs perform in their navigator function to coordinate patient care and ensure smooth pathways into and out of prison: 77% indicated that PMHTs perform ‘very well’ (*n* = 24) or ‘fairly well’ (*n* = 12). Respondents were asked to indicate how important follow-up by SPS is post remittal: 89% indicated that this is ‘very important’ (*n* = 32) or ‘important’ (*n* = 10). When asked how important follow-up from PMHTs is following inter-prison transfer, 90% responded ‘very important’ (*n* = 35) or ‘important’ (*n* = 7). See Table 3 for full PMHT responses.

**Table 3:** Characteristics of prison mental health services

Characteristics	*n* (%)
Category	
A	5 (10.6)
B	26 (55.3)
B, C^a^	7 (14.9)
Female	9 (19.1)
Prison same provider as local secure service	
Yes	23 (48.9)
No	24 (51.1)
Routinely ask about psychiatric detention	47 (100)
Routinely check care programme approach (CPA) status	47 (100)
Storage of CPA	
SystmOne	46 (97.9)
Other	1 (2.1)
Defined care pathway	
Yes and there is policy to support this	21 (44.7)
Yes but this is informal	19 (40.4)
No care pathway	2 (4.3)
No but we are developing one	1 (2.1)
Don’t know	3 (6.4)
Missing	1 (2.1)
Dedicated aftercare lead	
Yes	11 (23.4)
No	22 (46.8)
No – aftercare team	10 (21.3)
Don’t know	4 (8.5)
How well are prison mental health services meeting this responsibility?	
Very well	24 (51.1)
Fairly well	12 (25.5)
Moderately well	8 (17.0)
Not very well	2 (4.3)
Not well at all	0 (0)
Missing	1 (2.1)
How important do you think follow-up is from secure services?	
Very important	32 (68.1)
Important	10 (21.3)
Neutral	5 (10.6)
Low importance	0 (0)
Not at all important	0 (0)
Inter-prison follow-up	
Very important	35 (74.5)
Important	7 (14.9)
Neutral	4 (8.5)
Low importance	1 (2.1)
Not at all important	0 (0)

a Prisons housing both category B and C prisoners.

### Barriers and facilitators to effective discharge planning

Verbatim quotes are used in Tables 4–7 to illustrate key identified barriers and facilitators to effective discharge planning for those remitted to prison.

**Table 4:** Organisation and attendance at s.117 discharge planning meetings – barriers and facilitators to successful discharge planning and aftercare arrangements

Facilitators	Barriers
Secure psychiatric services	
‘Previously, it was common for the MH [mental health] In-reach team to join via teleconference, and more recently via Microsoft Teams (videoconference).’	‘We have had a couple of emergency remissions when it has not been possible to follow this process and we haven’t had a chance to have a s.117 meeting. All relevant information has however been emailed across subsequently in those cases.’
	‘Poorly organised with an unclear pathway as to who is to be invited and then it can be a case that insufficient staff attend and are done at short notice.’
	‘It is hit and miss as to whether they attend dependant of the prison. Some are better at nominating an attendee than others.’
Prison mental health teams	
‘We will not support or accept remission without attendance/involvement of s.117 discharge planning.’	‘This very rarely happens, unless a member the mental health team in prison, pressures the secure unit for time and dates, and also pressures their own managers to attend. Very often not enough time is given by the secure services in letting the mental health team to arrange someone to attend (this is a big problem).’
‘We have a transfers co-ordinator, who is an experienced mental health nurse, who acts as the main point of contact between prison mental health services and external providers.’	‘Hospitals do not routinely contact the PMHT [prison mental health team], nor do they seem to understand their responsibilities to communicate CPA [care programme approach]/s.117 information.’
‘We also invite a Custodial Manager from within our identified healthcare officer team to join this meeting to plan operational support to the patient on return, along with mental health.’	‘There have been cases where the MH team have not been invited by the hospital and we have been invited through another prison team.’
‘We are a Remand local and often patients are moving on very quickly, so we ask that prison involved in referral pathway is also invited to the s.117 meeting as this is where they will move on to after coming to us.’	‘We are sometimes (usually) invited to a 117 meeting, however, these may take place months before an individual’s transfer.’
	‘We attend these. We do however find if patients have returned to a local establishment before transferring to our prison, there is very little information available on SystmOne as sometimes the local establishment have not been involved in the s.117 meeting.’

**Table 5:** Nature of hand over information – barriers and facilitators to successful discharge planning and aftercare arrangements

Facilitators	Barriers
Secure psychiatric services	
‘We usually ask what information they need and at the very least provide up to date information on their mental state, physical health and treatment, including reports such as HCR-20, CPA [care programme approach] and tribunal reports.’	‘Feedback from prisons was that all the information provided in CPA documents, etc. was not necessarily useful for the prisons and therefore we added sections around information re: appropriateness to share a cell, safer custody requirements, etc.’
‘We ensure a discharge letter is available at the time of transfer, and we offer the patient time to talk with a staff representatives from the prison they are being transferred to.’	‘Hospital provides enough information when remission takes place, however this is not always shared with those who need to know once patient is back in prison services resulting in repeated requests for the same information by several different people.’
‘Our Prison Team Manager has a good relationship with the MSU [medium-secure unit] wards on site. We meet three times a week and have opportunity to keep each other informed of any change/details their team should be aware of. Local remand prisons are familiar with our systems and processes.’	
Prison mental health teams	
‘Documents are shared prior to 117 meeting. Identified new case manager meets the patient and secure hospital case manager and team Consultant reviews documents and agrees or declines transfer.’ ‘Full discharge summaries are received prior to remission. We work closely with our local secure hospital and have ongoing dialogue and information sharing in good time prior to the admission.’	‘We have found that some information needs chasing and that handover can be a little skewed when a hospital’s request for remission has mostly been driven by challenging behaviours that have caused difficulties in an in-patient setting. We have found it very hit and miss as to how well the individual is included in this process.’ ‘Currently the handover of information tends to take place verbally after such a meeting has taken place, which does not always provide opportunity to ask questions, manage expectations about what interventions/resources are available via the prison mental health service.’

**Table 6:** Aftercare planning/provision – barriers and facilitators to successful discharge planning and aftercare arrangements

Facilitators	Barriers
Secure psychiatric services	
‘Generally the aftercare is jointly planned so that both the hospital and prison are satisfied that the return is appropriate.’	‘There is no joint working to plan what care may look like on return to prison.’
‘It is helpful to follow up patients transferred to PRISON and for them to feedback to us if there is a further deterioration in the patients mental health state.’	‘Not enough [resources]. Due to this they are focussed on “fire fighting” immediate problems rather than longer term care planning.’
‘I have experienced good aftercare whereby probation have been involved and have a clear understanding of the needs of that individual and have fought to provide adequate care and treatment on returning to custody.’	‘It is difficult to comment on what resources the prison have. Part of the problem is that people tend to return to one of two specific prisons from our hospital and spend a short time there before being transferred to another one. So the vast majority of the time we do not know which prison they end up in.’
Prison mental health teams	
‘If we have attended the s.117 meeting then it can be discussed there what provision is appropriate for the patient and if and how this can be met in the prison environment.’	‘We very rarely conduct joint review. Unfortunately due to poor handovers, the prisoner may not always receive the recommended care as soon as they arrive.’
‘We have a discussion about what aftercare arrangements are: Including location when they return, what mental health and psychology support they need and identifying other partners who may need to be involved… we generally have sufficient resources for each individual to have an allocated PMHT [prison mental health team] practitioner and to be reviewed regularly. We have a psychology team who do some interventions and can do 1:1 if needed.’	‘One of the main issues that prison MH [mental health] teams face is that hospitals overestimate what they provide in terms of support/IPD [in-patient department] facilities/ability to sustain support when relapse occurs. Prison MH teams cannot utilise the MHA [Mental Health Act] 83 to administer treatment and so when a prisoner refuses treatment, relapse and deterioration can occur quickly. Hospitals can be slow to arrange assessments and admissions, leaving the prisoner in a state of deterioration when IPD facilities may not be resourced adequately.’
	‘Remand prison is inappropriate for most returners, they need to return to a slower stream establishment, which also avoids having to move twice if there sentence is to continue – we often find we can’t move them on; no one wants to take them.’

**Table 7:** Care programme approach (CPA) – barriers and facilitators to successful discharge planning and aftercare arrangements

Facilitators	Barriers
Secure psychiatric services	
‘We do not hold an extraordinary CPA prior to remission, although CPA meetings include pathway discussions which often precede a remission. If it is suspected that a remission may happen in the near future, the prison staff and in-reach team may be invited to attend the CPA.’	‘Process could be improved by having a dedicated person to liaise with and confirmation that all information relevant to the patient’s remission has been received.’
‘The CPA care plan for patients returning to prison is usually prepared with the assistance of the prison inreach team as they would be more aware of what treatment is and is not possible to provide within custody.’	‘The prison estate does not routinely engage in these plans. This is not a criticism but a reflection of the different clinical cultures.’
	‘There are certain patients – on longer term custodial sentences – for whom moves between prison and hospital become a part of their progress. For these individuals regular review meetings between prison and hospital healthcare settings would be beneficial.’
Prison mental health teams	
‘The CPA care plan is discussed at the section 117 meeting and any ongoing support needs will be continued here in prison. The care coordinator that takes over the care can sit down with the patient and draft a local care plan and how that care is going to be provided. The care plan is updated as the needs change or once the care objectives has been met.’	‘The plan is usually formulated with very little knowledge of prison environments and assumes that the MH [mental health] services in the prison are the same as secure hospitals which of course they are not. We are trying to arrange some insight visits for SPS [secure psychiatric services] staff.’
‘We are in the process of developing the CPA process due to the changing population and function of the prison.’	‘Not usually received, interim care plan developed on return until CPA takes place and further care plan developed.’ ‘We are not great at reviewing them as clients are rarely with us for long before being transferred/released and so energy then goes into release planning/transfer handovers of care.’
	‘The service user does not wish to engage in/contribute to the formulation of the care plan whilst in prison.’
	‘It is not always evidenced that service users get copies of these [care plans].

#### (a) Organisation and attendance at s.117 discharge planning meetings

All SPS noted the importance of PMHT attendance at s.117 discharge planning meetings, describing routine invitation, with attendance recorded as good but not consistent. Attendance from custody staff was described as less consistent. Securing PMHT attendance was described as sometimes challenging, particularly if the remittal prison destination was not identified or secured, indicating that s.117 meetings are held prematurely in some instances. Others described how meetings are sometimes repeated for PMHTs to attend. One PMHT reflected that some s.117 meetings take place so far in advance of remittal that their knowledge of the patient is unlikely to be accurate following transfer. Some respondents explained that delays can be due to prison refusal to accept a particular patient where they have knowledge of their presentation during a previous stay at their site. The opposite was also described, where remittal is an emergency due to either patient high-risk behaviours or unexpected receipt of a custodial sentence at court. In these instances, it is not possible for the s.117 meeting to take place in advance of remittal. Many PMHTs reported inconsistent invitation to s.117, describing how they had learned of an arranged s.117 meeting via custody staff and have had to ‘chase’ SPS to secure an invite. Similarly, one PMHT described instances of remittal warrants being signed by the prison and the PMHT not being aware of the patient being remitted until they were housed on the prison wing.

While the majority of SPS described their organisation of s.117 meetings favourably, others described how at times, these are disorganised and they are unclear on whom to invite, leading to there being insufficient time for the PMHT to arrange attendance. All PMHTs referred to the importance of s.117 attendance, with some describing how they will refuse to accept a patient for remittal if this has taken place in their absence, and many describing how they request that a custodial manager attends the meeting with them. One PMHT described having a dedicated transfer co-ordinator employed by their trust who monitors patients who may be approaching remittal, to ensure they are able to contribute to discharge planning in advance of the s.117 meeting. One PMHT described that due to them being a ‘local’ remand prison (to which sentenced patients are remitted before transfer to an appropriate prison), they request that the subsequent transfer prison is also invited. This was highlighted as important by another PMHT who shared their experience of receiving no information from a patient’s remittal prison following inter-prison transfer. Both SPS and PMHT teams described how accessing technology to attend s.117 meetings virtually has increased PMHT ability to attend meetings.

#### (b) Information handover

SPS reported how they communicated well with PMHTs to handover information about a patient’s treatment, including discussing risk assessments and discharge summaries at s.117 meetings. Multiple SPS reported working with PMHTs to adapt their hand over documentation to ensure that the nature of information is relevant to the prison environment. Outside of the s.117 meeting, SPS described how they are able to provide extensive information electronically to PMHTs; however, it is not always clear to whom this information should be provided, nor is it always shared among the clinical team, resulting in repeated requests for the information. Successful information transfer was described in some cases as due to close relationships between SPS and PMHTs, together with the presence of psychiatrists who work across both services, enabling effective communication. One SPS site reported meeting with the local PMHT three times weekly to discuss patients who were transferred, discussing progress and plans far in advance of remittal.

Many PMHTs reported receipt of adequate information but reported that it would be more appropriate to receive this prior to the s.117 meeting, because the verbal hand over during the meeting leaves minimal time to ask questions or get to know the patient. Due to either emergency transfers or the absence of timely communication, PMHTs reported receiving information handover after the person had been remitted resulting in the patient not always being transferred to the most appropriate setting within the prison. One service commented that information is often ‘skewed’ in instances of emergency transfer, resulting in them having to seek further information following the patient’s return.

#### (c) Aftercare planning/provision

Planning for aftercare and the degree of aftercare provision were described as variable by responders from both surveys. There were reports of strong relationships between SPS and PMHTs where meaningful joint aftercare planning is possible, with one service reporting how this also includes a member of the probation team to plan for release post remittal. It was described that planning in advance of the s.117 meeting is preferable. There was also one report of a very well-resourced PMHTs, where patients have access to one-to-one psychology upon remittal if required. However, most responses indicated that PMHTs are generally under-resourced and are unlikely to meet the needs of remitted patients. PMHT services were described as over stretched, where focus is on ‘firefighting’ immediate need as opposed to long-term care planning. PMHTs described how SPS often overestimate what they can provide for remitted patients, leading to inappropriate returns and patients who deteriorate and relapse. PMHT clinicians described how this was due to the issues described in the foregoing sections: poor or minimal relationship between services, failure to engage PMHTs before or with the s.117 process and limited handover. There was little reference to SPS follow-up across responses from either survey. Some SPS respondents described a 72 h or 7 day follow-up phone call. One service stated that they are a private healthcare provider and are therefore not funded to enquire about patient progress following remittal. PMHTs described how it is difficult to re-engage SPS to reassess patients who are observed to deteriorate, leaving them with inadequate access to resources. Both SPS and PMHTs described how the current policy of remitting patients to the ‘local remand’ prison is inappropriate and often results in loss of aftercare due to limited handover or limited access to resources. No PMHT reported having a policy/procedure in place to formally discontinue s.117 aftercare.

#### (d) CPA and continuation beyond remittal

There were few examples of collaborative CPA development across SPS and PMHTs, although one SPS site described inviting a PMHT to routine CPA meetings when remittal is indicated; this was deemed as important for gaining an understanding of the care available within the prison environment. In another SPS site, the patient’s final CPA meeting was described as held in conjunction with the s.117 meeting. Some described non-engagement from PMHTs. Most responses from both SPS and PMHTs indicated that the latest CPA report/plan was included within the discharge summary documentation that is provided to PMHTs during or following the s.117 meeting. In these instances, while this was described as a rich source of information by PMHTs, the resources available to them are rarely considered to be meeting patients’ support needs. PMHT clinicians described how, following remittal, they routinely develop an interim care plan that remains in place until they have capacity to develop a CPA that is suited to the prison environment. There were some reports of PMHTs not routinely receiving handover of CPA from SPS.

There was very little reporting on continuation with CPA post remittal. Some PMHTs based at remand prisons described how this was due to the nature of the environment, with plans often going unreviewed due to inter-prison transfer or release. PMHTs reported that CPA meetings are not usually a priority. These meetings were described as difficult to arrange due to other clinical commitments, and there were examples of patients not wishing to engage with care planning once returned to prison, or not receiving copies of their care plan.

## Discussion

This is the first study to investigate discharge planning and aftercare arrangement practices in SPS and PMHTs in England and Wales; survey responses were received from 72% of contacted SPS and PMHTs. Overall, the data presented above highlight the fact that effective discharge planning between SPS and PMHTs is not currently taking place nationally, and in some areas is not conducted in line with good practice guidance.^2^ Respondents described a wide range of barriers and facilitators that impact on engagement with the s.117 discharge planning process, information handover, planning for aftercare and delivery of aftercare via the CPA process.

### System communication

A key factor described across all areas was the impact of the nature of the relationship between SPS and PMHTs. Successful organisation of s.117 meetings and purposeful handover and aftercare planning were reported as present in services where relationships between SPS and PMHTs were described as ‘positive’ and ‘well established’, and respondents provided examples of local initiatives that foster these relationships. This included arrangement for weekly meetings between SPS and the local reception PMHT, where patient progress is discussed, and remittal is planned in advance of s.117 meetings. The respondent described how this also allows for advance engagement of custody staff and ensures prison representation at prospective s.117 meetings. There were also examples of SPS having consulted with local PMHTs to adapt discharge planning documentation, to ensure that care plans are relevant to the prison environment and feasible to deliver within PMHT provision. In areas where poor or minimal relationships between SPS and PMHTs were described, this was accompanied by a lack of robust processes and practices outside of good clinical practice guidance. This included inconsistent invitation and attendance at s.117 meetings, with instances of PMHTs not being aware of a remitted patient until they were housed in an ordinary location. Limited hand over was also described, alongside unfeasible aftercare plans developed by SPS in isolation. To meet the needs of patients at this important transition point, the interface between SPS and PMHTs requires improvement.

SPS and PMHT relationships were also described as contingent on the healthcare provider commissioned to deliver the PMHT, with positive relationships described across sites having the same NHS provider. Almost half of PMHT respondents reported that they shared the same NHS provider as their regional SPS. At these sites, a SPS responsible clinician was also often the visiting psychiatrist at the local reception prison, and therefore was able to ‘manage’ the patient’s return to prison. Likewise, in one region, all PMHTs shared the same NHS provider, and, across these teams, an experienced clinician was employed as a ‘transfer coordinator’ and with had oversight of all patient transfers and remittals. This was the only reported example of this clinical role nationally. This role was presented as performing a similar function to that proposed in the White Paper *Reforming the Mental Health Act*, published in January 2021,^23^ where the review called for the introduction of an independent role to manage patient transfers and remittal. In response to this, the government has pledged that, while it will remain for the Secretary of State for Justice to formally approve the transfer of a prisoner to SPS and subsequent remittal, a new designated role will be established.^24^ It is anticipated that this role will be independent of the health or criminal justice system and will help to ensure that institutional barriers are overcome and that the patient’s needs are put first. The role is described as being similar to the Approved Mental Health Professional role in civil settings, where the professional will be involved from the point of initial referral for access assessment, through to liaising with the range of organisations that may be needed to support someone’s return to prison. It is envisaged that this role may sit within NHS England and NHS Improvement but will be separated into teams that are responsible for commissioning/provision of SPS beds. The specifics of this role remain under national consultation, and it is important that decision-makers consider how this role will embed into current remittal policy.

At present, patients returning to custody from SPS return to the local ‘reception’ prison in that area (unless there are exceptional services to prevent this or they are a category A prisoner,^2^ after which they undergo inter-prison transfer, returning to their originating prison or elsewhere in their region if subject to recategorisation. Inter-prison transfer is a key transition point for loss or denial of aftercare, with almost half of those transferred not being handed over between PMHTs.^11^ To ensure that patient benefits intended by this role are maintained, a review of the local reception prison policy should be conducted, and consideration should be given to the professionals’ role in this transition.

### Return and aftercare provision

The past 20 years has seen a focus on transfer of prisoners to SPS,^25^ with little attention given to the remittal care pathway. The 2021 good practice guidance introduced for the first time a remittal time scale target: a maximum of 14 days between a responsible clinicians decision to remit and remittal to prison^2^ within which there are associated time scales for convening a s.117 meeting, agreeing a care plan, and issuing a warrant. While a speedy remittal is advantageous in terms of increasing SPS bed capacity, poor management of this transition is associated with relapse, re-referral and readmission to SPS.^11^ It is our concern that this current guidance does not account for the difficulties faced by SPS and PMHTs outlined in our analysis and that a tight timescale may hinder even further effective remittal and planning for appropriate aftercare services in the prison estate. We are aware that NHS England are currently working to develop a programme plan for a national mental health pathway across health and justice, within which there is a focus on systems/processes related to transfer between in-patient mental health services and remittal to prison. We advocate for this work to be extended to consider the mental health provision available in prison, including the nature of aftercare services available following remittal.

There are vast differences observed across PMHTs in terms of design and resources available, with varying caseloads and insufficient staffing.^26^ In this study respondents described how the resources available to PMHTs are not adequate to meet patients’ support needs following remittal, and that the use of CPA for these patients is neither practical nor widespread. Less than half of the PMHTs reported routinely checking a patient’s CPA status. In general CPA practice in prisons lacks standardisation and consistency and is not currently meeting the expected standard of care. The Quality Network for Prison Mental Health Services (QNPMHS) has devised national standards for effective delivery of PMHTs,^19^ with earlier iterations having presented four standards relating to delivery of CPA in prisons.^27^ Between 2016 and 2018 compliance with these standards was assessed as low, particularly in relation to ensuring that patients are fully involved and consulted about each stage of the CPA process.^28^ The QNPMHS has since conducted a national consultation to inform the development of guidance on planning of effective mental health in prisons in relation to the CPA.^29^ It concluded that for PMHTs to be able to achieve the expected standard of care and empower patients to be involved in their recovery, services and partner agencies would require additional resource, dedicated administrative support, improved training, streamlined processes and standardised templates. If delivered effectively, the CPA has the potential to enhance care delivery and improve outcomes for people in prison.

Significant reform is required to provide people remitted to prison from SPS with a package of aftercare that is suitable to their needs and that follows them throughout their pathway and reintegration into the community. A well-resourced PMHT would enable more effective release planning and continuity of care, thereby reducing the risk of relapse or deterioration of mental health and ensuring that individuals continue to receive necessary support. This study highlights a significant gap in the involvement of local authorities in discharge planning and aftercare arrangements for individuals remitted to prison from SPS. Neither SPS nor PMHT representatives reported successful engagement with social care providers during s.117 meetings or in the coordination of ongoing or discontinued aftercare. This underscores a pressing national need to establish formal communication channels and protocols to ensure that local authorities play an active role in s.117 meetings, discharge planning and aftercare arrangements. Additionally, no PMHT reported having formal policies or procedures in place to discontinue s.117 aftercare. Since PMHTs and local authorities share a joint statutory duty to provide aftercare for as long as it is needed, there is a critical need to clarify how changes in aftercare needs are assessed and agreed upon in prisons. The current lack of clarity on patient involvement in this process further compounds the risk of breaching statutory s.117 obligations. To meet these duties, joint decisions must be recorded, justified, and based on appropriate assessments of a patient’s aftercare needs, with plans that clearly outline how those needs will be addressed. Therefore, PMHTs and local authorities should collaborate to develop robust policies and procedures for assessing and documenting changes in need, ensuring statutory compliance and consistent decision-making. Addressing these shortcomings is essential to achieving equivalence in health and social care outcomes for individuals in prison compared with those in the community.

NHS England health and justice is currently conducting a national consultation to develop an integrated mental health pathway between prisons and SPS. The findings of this study offer valuable insights to guide the development of this pathway for individuals remitted to prison. Specifically, we recommend national consideration of the following priorities: (a) enhancing discharge planning and aftercare practices, (b) improving system communication and collaboration, (c) establishing a new clinical role to oversee transitions, (d) addressing resource disparities within PMHTs, (e) standardising CPA practices across prisons and (f) engaging local authorities in aftercare provision. By integrating these recommendations, NHS England can establish a comprehensive integrated mental health pathway that ensures continuity of care, reduces inequities, and improves outcomes for one of the most vulnerable patient populations.

### Strengths and limitations

This study provides the first national-level overview of discharge and aftercare planning for those returned to prison from SPS in England and Wales. The findings can be utilised by NHS England and NHS Improvement to inform the development of the national mental health pathway across health and Justice. Although this study received a 72% response rate, it does not represent the experience of all eligible services who were contacted for participation. Likewise, we had a higher response rate from PMHTs than from SPS meaning that the findings may be skewed towards the PMHT perspective. It is also important to note that the survey was completed by just one clinician at each site, and therefore their views may not be representative of the whole clinical team.

Our current understanding of the remittal care pathway in England and Wales would benefit greatly from exploration of patient experiences and perspectives of how discharge planning affects individuals transitioning between SPS and prison.

## Data Availability

The data that support the findings of this study are available from the corresponding author, S.L., upon reasonable request.
